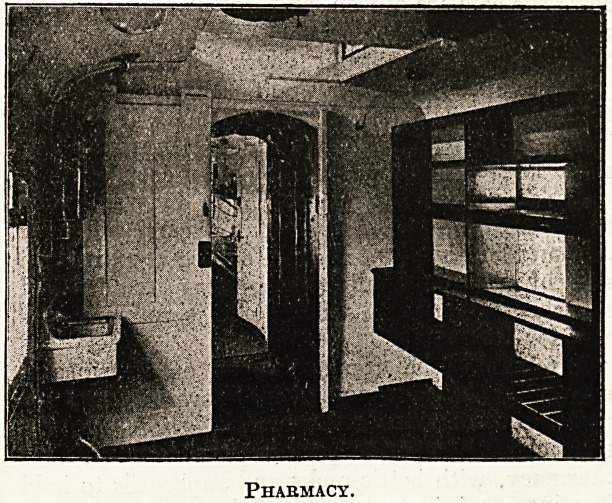# Organisation of a Red Cross Train: The Transit by Rail

**Published:** 1915-04-17

**Authors:** 


### ORGANISATION OF A RED CROSS TRAIN.

### The Transit by Rail.

By AN R.A.M.C. OFFICER IN CHARGE.

An ambulance train is generally constructed by the railway company from the rolling stock which is in ordinary use. The long express luggage-vans have been found to be the most easily adaptable for the purpose. In the case of a typical ambulance train in this country, for the transport of 100 cot cases, ten coaches are provided—five as wards; one as kitchen; one for treatment room, dispensary, and

Ward with Cots Turned Up.

store room; and three for the accommodation of the staff.

Each ward can accommodate twenty lying cases in cots; the cots are of iron, and arranged in two tiers, one above the other, fixed on each side of the carriage, with a gangway down the middle. The cots have spring bottoms and can be turned up against the walls and fixed in that position. The floors are covered with linoleum; walls enamelled; and a lavatory arranged at the end of each ward.

Lighting is by gas or electricity; and ventilation by extractors on the roof of the carriage or by windows at the sides. A large sliding door on each side of the ward allows the easy removal of stretcher cases. The whole train is connected by corridor fastenings.

A room suitable for operations or dressings is arranged with water supply, lead-covered floor, and rounded corners to facilitate disinfection. In the same coach a separate part is partitioned off as a pharmacy, with bottle-racks and cupboards to hold dressings and instruments. Other rooms are arranged for storage of linen, pillows, etc., and for dirty or infected bedding; and one room is fitted-up as an office. The coaches for the staff are of the corridor type. Steam heating by radiators is arranged throughout the train.

The accommodation of an ambulance train in this country, as already explained, is for 100 cot cases; on the Continent this number has been much exceeded, also the *personnel* differs very much. In England, the usual staff consists of one medical officer in charge of the train, with two nurses and ten non-commissioned officers and men; in addition, two cooks are provided. The distribution of the non-commissioned officers and men can be as follows: one sergeant in charge of three wards, and a corporal in charge of two. One orderly, who must always be present, in each ward, this leaves three orderlies for general duties, such as distributing food, cleaning-up, and fetching dressings.

### Loading-up from the Hospital Ship.

As soon as notice has been received that a hospital ship is coming in at the port of disembarkation, notice is sent to the officers in charge of the trains required, giving the number of patients to be taken, class of cases, whether cot or walking cases, and destination. If the journey is of any length, rations have to be drawn for the patients. The trains are then drawn up as near to the hospital ship as possible, and in the order in which they are to depart.

Cots are prepared with mattresses, sheets, blankets, and pillows for the number of cot cases expected. When a number of cases can sit up, one or more wards should be prepared for them; the lower cots alone are used, the upper ones being turned up and fixed against the side of the ward. The mattress of the upper cot is arranged upright at the back of the lower cot as a rest for the men

Ward with Mattresses and Pillows.
(Note roof ventilators.)

sitting, and sheets are put over both mattresses to prevent them from being soiled.

Where a large number of sitting cases have to be taken, extra corridor carriages can be added for their accommodation.

The rations which have been drawn are prepared by the cooks ready for distribution to the wounded as soon as the journey commences. If early in the morning, a meal of bacon, bread, and tea is first served; in the middle of the day, a dinner of meat, vegetables, and bread; Bovril, arrowroot, cocoa, or milk can also be got ready for patients unable to take the ordinary fare. As the number of wounded carried may be anything up to 200 cases, the cooks have a busy time.

### The Art of Unloading.

As soon as the hospital ship has been berthed, unloading commences. The wounded unable to walk are carried on stretchers by a separate squad of R.A.M.C. men to the trains; the large sliding doors in the wards are opened and the stretchers handed over to the train orderlies, who then lift the patients on to the cots and return the stretchers to the unloading party.

A Corner of the Operating Room.

The best way to unload a helpless patient from a stretcher is as follows: in addition to the stretcher-bearers three orderlies are required, one for head and shoulders, one for middle of body, and one for the legs; the stretcher is rested on the edge of the cot; the patient is raised off the stretcher, which is then dropped down vertically; the orderlies can then carry the patient on to the cot. No attempt should be made to reach across the stretcher holding the patient, as it is bound to jar him and may cause injury.

The arrangement of the wounded in the wards is of importance. It is best, if possible, to have the cot cases in the wards nearest the kitchen, the sitting cases being furthest away. Serious cases, such as men wounded in the lower limbs, or otherwise helpless, should be put in the lower cots. It is also best to arrange the wounded so that the injured limb may be outermost. The cots should be filled from the end of ward furthest from the door, as this avoids carrying stretchers past the patients already in the cots, a slight knock to a seriously wounded man might cause much pain.

When the ward is full, doors are closed, and the nurses come round and attend to dressings and the comfort of the men. A little care in arranging pillows or cushions may make an enormous difference in the comfort of a wounded man travelling in a train. In the early days of the war, when the wounded were brought down from the Aisne in railway trucks, the journey often taking several days, it was delightful to find how the comfort on the ambulance train improved their condition.

The journey commenced, a meal is served, wounds attended to, and cigarettes or tobacco handed round; the patients soon make themselves comfortable and enjoy their new surroundings. A nominal roll of all patients has to be made, with notes of their injuries.

On journeys of any length the train is stopped at one or more rest stations; cakes, fruit, tea, and cigarettes are passed into the train by ladies of the Voluntary Aid Detachments, and also some of the ladies are allowed into the wards to distribute the good things themselves. The wounded thoroughly enjoy the attention paid to them, and it is wonderful how they cheer up. The rest stations have undoubtedly contributed very much to their comfort.

On arrival at the destination, the officer in charge of the train arranges with the officer in charge of the hospital to which the wounded are going the order in which he wishes them removed, as regards cot cases or walking cases. The ambulances or motor-cars are arranged by the Voluntary Aid Detachments as near as possible to the train. The stretchers are then passed into the wards, loaded with wounded by the train orderlies, and passed out again to the Voluntary Aid Detachments. The order in which the wounded are unloaded should be in the reverse order to that of loading—*i.e.*, cases should be taken from nearest the door first.

### The Return Journey.

When the train is empty and the return journey is commenced the work of the orderlies has by no means ceased. Sheets and blankets have to be removed from the beds, soiled sheets put away to exchange for clean ones on arrival at the port of disembarkation. Blankets are shaken and folded up, cots turned up against the sides of the ward, and the wards first brushed through, and then washed with soap and disinfectant. The paint must then be washed over, the beds made, and everything prepared for a new load of wounded on arrival at the port again. When everything is shipshape the tired staff turn in to sleep until awakened by the shunting of the train in the docks.

Pharmacy.

## Figures and Tables

**Figure f1:**
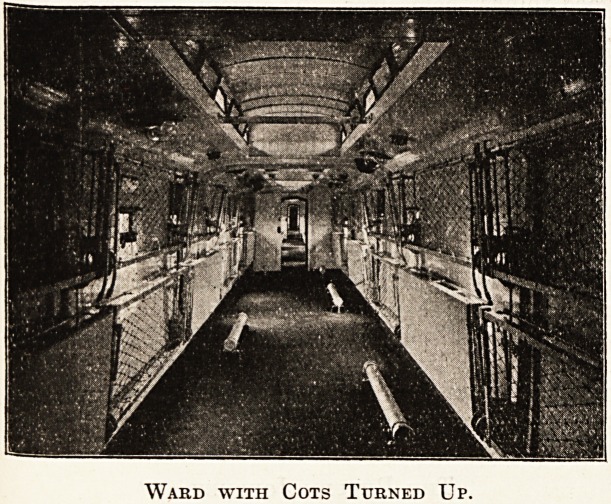


**Figure f2:**
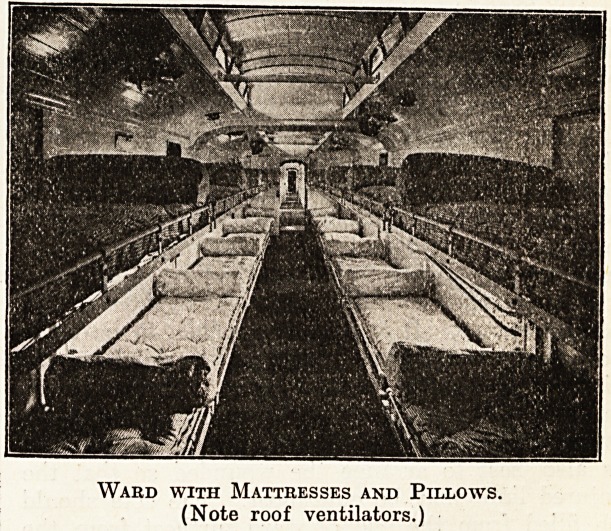


**Figure f3:**
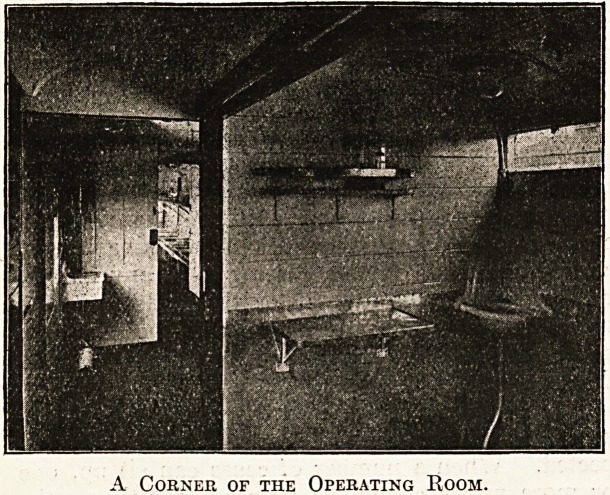


**Figure f4:**